# Concurrent optimization of fracture toughness, thermal conductivity, and tribological behavior in C_f_/Si_3_N_4_ composites via phase driven selection

**DOI:** 10.1038/s41598-026-44244-7

**Published:** 2026-03-28

**Authors:** Saeed Hoseinzadeh, Mohammad Reza Loghman Estarki, Ali Ghasemi, Saeed Zahabi, Gholamreza Gordani, Ehsan Mohammad Sharifi

**Affiliations:** Department of Materials Engineering, Malek Ashtar University of Technology, Isfahan, Iran

**Keywords:** C_f_/ Si_3_N_4_ composite, Coefficient of friction, Spark plasma sintering, Thermal conductivity, Microstructural design, Silicon nitride polymorphs, Engineering, Materials science

## Abstract

Carbon fiber–reinforced silicon nitride (C_f_/Si_3_N_4_) composites were fabricated by spark plasma sintering (SPS) using α-, β-, and γ-Si_3_N_4_ powders to clarify the influence of the initial Si_3_N_4_ phase on microstructural evolution and functional properties. The results show that the starting phase significantly affects densification behavior, phase transformation, and mechanical and tribological performance. The composite derived from α-Si_3_N_4_ achieved the highest relative density (96.53%) and exhibited an optimal balance of fracture toughness (10.87 MPa m^0.5^), thermal conductivity (66 W/m K), and stable friction behavior (COF ≈ 0.46). This performance is attributed to the in-situ formation of a self-reinforced β-Si_3_N_4_ microstructure during the α → β phase transformation, which promotes crack deflection, crack bridging, and effective load transfer in synergy with carbon fibers. In contrast, β- and γ-Si_3_N_4_–based composites showed lower densification or excessive hardness associated with increased porosity and secondary phase formation. These findings demonstrate that controlling the initial Si_3_N_4_ phase provides an effective microstructural design strategy for developing high-performance C_f_/Si_3_N_4_ composites for thermostructural applications such as aerospace brake discs.

**Supplementary Information:**

The online version contains supplementary material available at 10.1038/s41598-026-44244-7.

## Introduction

Silicon nitride (Si_3_N_4_)–based ceramics are widely recognized as promising materials for high-temperature and tribological applications due to their excellent mechanical strength, thermal stability, and wear resistance. These attributes make Si_3_N_4_ an attractive candidate for demanding thermostructural components such as aerospace brake discs, where a delicate balance between high fracture toughness, stable friction behavior, and effectiveological applications due to their excellent mechanical strength, thermal stability, and wear resistance. However, the inherent brittleness of monolithic Si_3_N_4_ limits its damage tolerance under severe mechanical loading and rapid thermal cycling, restricting its direct application in advanced braking systems^1–5^.

Currently, carbon fiber–reinforced silicon carbide (C_f_/SiC) composites represent the state-of-the-art materials for high-performance brake discs in aerospace and motorsport applications. C_f_/SiC systems exhibit high specific strength high-performance brake discs in aerospace and motorsport applications. Cf/SiC systems exhibit high specific strength, good thermal conductivity, and excellent oxidation resistance, good thermal conductivity, and excellent oxidation resistance, particularly when fabricated via chemical vapor infiltration (CVI) or polymer infiltration and pyrolysis (PIP). Nevertheless, their relatively moderate fracture toughness, sensitivity to thermal shock under extreme conditions, and high manufacturing cost remain persistent challenges, especially for next-generation aerospace braking systems^6–9^.

In this context, carbon fiber–reinforced silicon nitride (C_f_/Si_3_N_4_) composites have emerged as a compelling alternative. The incorporation of carbon fibers into a Si_3_N_4_ matrix can significantly improve fracture toughness and thermal shock resistance through mechanisms such as crack deflection, crack bridging, and fiber pull-out, while simultaneously enhancing tribological stability. The final performance of C_f_/Si_3_N_4_ composites, however, is strongly governed by densification behavior, fiber–matrix interfacial bonding, and microstructural evolution during sintering^10,11^.

Among the final performance of C_f_/Si_3_N_4_ composites, however, is strongly governed by densification behavior, fiber–matrix interfacial bonding, and microstructural evolution during sintering^10,11^. Among the parameters controlling microstructural development, the initial phase of the Si_3_N_4_ powder—α, β, or γ—plays a decisive role. The well-known α → β phase transformation during the parameters controlling microstructural development, during sintering promotes the formation of elongated β-Si_3_N_4_ grains, which act as an in situ self-reinforcement network and substantially enhance fracture resistance. In contrast, β-Si_3_N_4_ powders generally exhibit limited grain growth, while γ-Si_3_N_4_ powders, due to their amorphous nature and finer particle size, may hinder effective densification despite promoting high hardness^2–7^.

Spark plasma sintering (SPS) offers distinct advantages for the consolidation of Si_3_N_4_-based composites, including rapid heating rates, short dwell times, and enhanced mass transport, which collectively enable high densification at relatively lower sintering durations. When combined with appropriate oxide sintering aids (e.g., Al_2_O_3_ and Y_2_O_3_), SPS can promote liquid-phase-assisted densification while suppressing thermal decomposition of Si_3_N_4_^2,7^.

Despite extensive research on monolithic Si_3_N_4_ ceramics and C_f_/SiC brake materials, a systematic and comparative investigation of C_f_/Si_3_N_4_ composites fabricated from different initial Si_3_N_4_ phases under identical SPS conditions remains notably limited. Therefore, the present study aims to elucidate the influence of α-, β-, and γ-Si_3_N_4_ starting powders on phase evolution, microstructural development, densification behavior, and the resulting mechanical, thermal, and tribological properties of C_f_/Si_3_N_4_ composites. By establishing clear phase–microstructure–property relationships, this work introduces a phase-informed microstructural design strategy that moves beyond conventional composition-based approaches, offering a viable alternative to SiC-based systems for design strategy that moves beyond conventional composition-based approaches, offering a viable alternative to SiC-based systems for advanced aerospace braking and thermostructural applications^8–14^.

## Experimental

### Materials

**Fig. 1:**
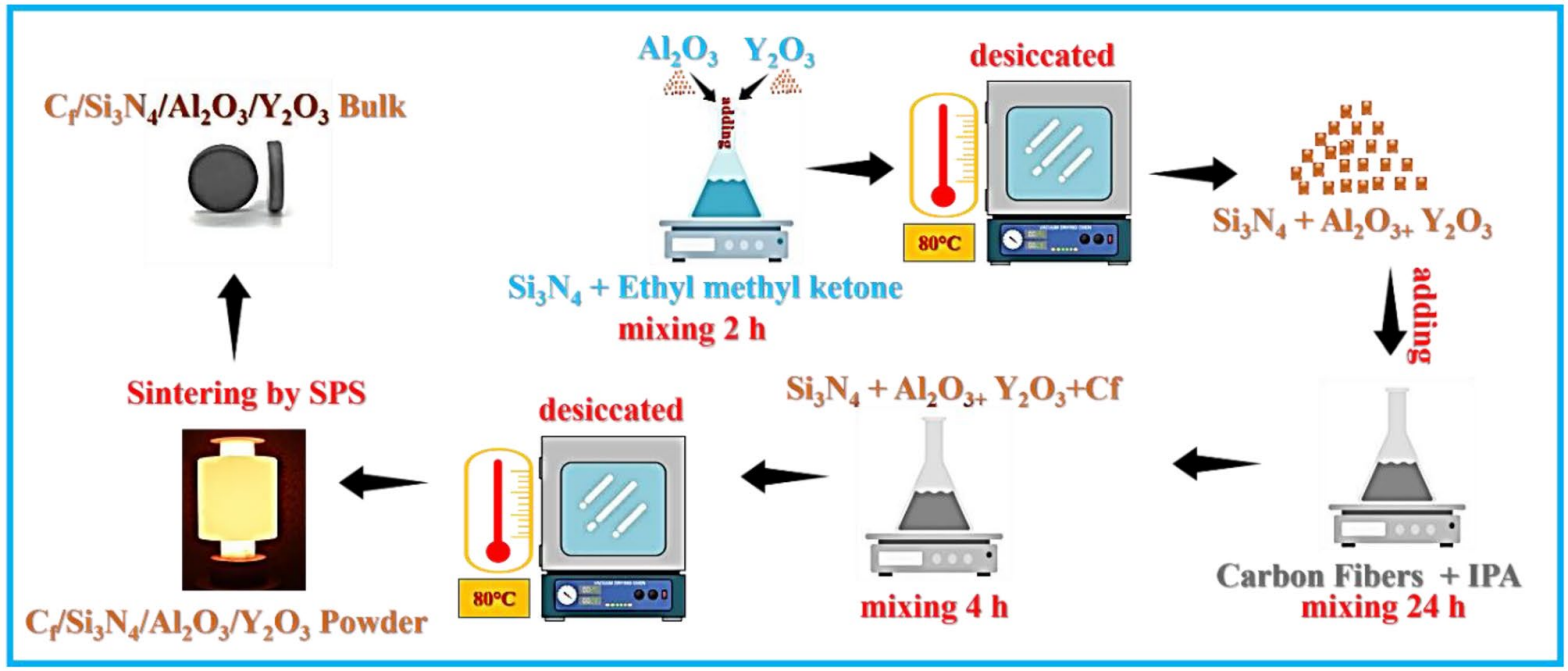
Schematic of the C_f_/Si_3_N_4_/Al_2_O_3_/Y_2_O_3_ composite bulk production process .

Commercial silicon nitride (Si_3_N_4_) powders with α-, β-, and γ-phases were purchased from US Research Nanomaterials, Inc. (Houston, USA). It should be noted that X-ray diffraction (XRD) analysis revealed the presence of approximately 10 wt.% β-Si_3_N_4_ in the as-received α-Si_3_N_4_ powder. In addition, the commercially labeled γ-Si_3_N_4_ powder was found to be predominantly amorphous, in agreement with the supplier’s specifications and XRD results (Figs. S1 and S2, Supplementary Information) (Fig. 1).

Aluminum oxide (Al_2_O_3_, 99.5% purity, average particle size ~ 150 nm, mixed hexagonal/monoclinic structure; Fig. S2b) and yttrium oxide (Y_2_O_3_, 99.9% purity, average particle size ~ 50 nm, rhombohedral structure; Fig. S2c) were used as sintering aids. Continuous carbon fibers (T300 grade, average diameter ~ 6 µm; Fig. S3) were supplied by TAM Co. (Seoul, Korea) and cut into 5 mm lengths using a precision fiber cutter prior to composite fabrication. Methyl ethyl ketone (MEK) and isopropyl alcohol (IPA) (Merck & Co.) were employed as dispersion media during powder processing.

The morphology and chemical composition of the as-received Si_3_N_4_ powders (α, β, and γ) and carbon fibers were characterized using field-emission scanning electron microscopy (FESEM) equipped with energy-dispersive spectroscopy (EDS). As shown in Fig. S4 (Supplementary Information), the α- and β-Si_3_N_4_ powders exhibited a semi-spherical morphology with particle sizes in the range of 500–600 nm, whereas the γ-Si_3_N_4_ powder consisted of finer particles with sizes of approximately 200–300 nm. In addition, the α-Si_3_N_4_ powder contained a minor fraction of rod-like particles with diameters of ~ 250 nm and lengths of ~ 1 µm. EDS analysis confirmed the high purity of all powders, with no detectable impurities above 0.5 wt.% (Fig. S4).

## Fabrication of the Cf/Si3N4/Al2O3/Y2O3 nanocomposite

The composite powders were prepared through a three-step dispersion process. In the first step, 1.5 g of Si_3_N_4_ powder was dispersed in 250 mL of MEK and homogenized for 2 h using mechanical stirring and ultrasonication. In the second step, 0.06 g of Al_2_O_3_ and 0.12 g of Y_2_O_3_ were added to the Si_3_N_4_ suspension and further dispersed for 1 h. The resulting slurry was then dried in an oven at 80 °C to remove the solvent.

In the third step, carbon fibers (20 wt.%, initial length 5 mm) were dispersed in IPA using ultrasonic agitation for 24 h to ensure uniform separation. The fiber suspension was subsequently mixed with the pre-dispersed Si_3_N_4_/Al_2_O_3_/Y_2_O_3_ powder and further homogenized for 4 h by combined magnetic stirring and ultrasonication. Finally, the mixed slurry was dried at 80 °C until complete solvent evaporation, yielding homogeneous Cf/Si_3_N_4_/Al_2_O_3_/Y_2_O_3_ composite powders.

The resulting composites based on α-, β-, and γ-Si_3_N_4_ precursors were denoted as Sample 1 (C_f_/α-Si_3_N_4_), Sample 2 (C_f_/β-Si_3_N_4_), and Sample 3 (C_f_/γ-Si_3_N_4_), respectively.

## Sintering of the Cf/Si3N4/Al2O3/Y2O3 nanocomposite powders

The prepared composite powders were consolidated using a SPS system equipped with a graphite die. A graphite foil with a thickness of 0.3 mm was placed between the powder compact and the die to prevent adhesion. Sintering was conducted under vacuum (~0.1 Torr) at 1850 °C for 20 min, applying a uniaxial pressure of 70 MPa and a heating rate of 200 °C min^−1^.

## Characterization tests

The phase composition of the starting powders and sintered composites was determined by X-ray diffraction (XRD) using a PANalytical X’Pert Pro MPD diffractometer with Cu–Kα radiation (λ = 1.542 Å), operated at 40 kV and 30 mA, with a step size of 0.05°. The average crystallite size was estimated using the Scherrer equation^15^:

$$D = k\lambda /\beta \cos \theta$$

where λ is the wavelength (Å), k is the shape factor (~ 1), θ is the diffraction angle, and β is the full width at half maximum (FWHM, radians).

Morphology and microstructure of the powders and sintered ceramics were examined by field-emission scanning electron microscopy (FESEM, QUANTA PEG 450) equipped with energy-dispersive spectroscopy (EDS) for chemical composition assessment.

The bulk density of the sintered ceramics was measured via the Archimedes method according to ASTM C373^16^. Vickers hardness measurements (ASTM C1327-15)^17,18^ were conducted at five different locations with a 9.8 N load (1 kgf) and 15 s dwell time.

Fracture toughness (*K*_IC_) was estimated from Vickers indentation using both Anstis et al. and Niihara models to account for possible crack types (Palmqvist vs. median/half-penny)^19,20^. Crack lengths were measured from FESEM images (Fig. S5), and *K*_IC_ was calculated by:

1 $$K_{{{\mathrm{IC}}}} = 0.0{16}\left( {{\mathrm{E}}/{\mathrm{Hv}}} \right)^{{{1}/{2}}} ({\mathrm{P}}/{\mathrm{c}}^{{{3}/{2}}} )$$

where E is the Young’s modulus, c is crack length Hᵥ is the Vickers hardness (Hᵥ (GPa) = 1.854 F / (2a)2), and P is the indentation load.

The fracture toughness (*K*_IC_) values reported herein were derived from the Vickers indentation method. It is critically acknowledged that this technique provides an estimate of 'indentation fracture resistance’ rather than an absolute measure of fracture toughness, as it is sensitive to crack morphology (Palmqvist vs. median/radial systems) and residual stress states, and may not fully capture the toughening contributions of long fibers in a composite^19–21^. Therefore, the presented *K*_IC_ values should be interpreted primarily as a consistent comparative metric to evaluate the relative performance among the three composite variants (α-, β-, and γ-derived) processed and tested under identical conditions. The observed trends are further corroborated by flexural strength tests and microstructural analysis.

Tribological testing was performed using a pin-on-disk setup (ASTM G99-17)^22^ at a 5 N normal load and 0.5 m/s sliding speed. This load was selected based on Hertzian contact analysis for a sphere-on-flat configuration^22,23^ to reproduce realistic contact stress (~ 1.2 GPa) at aerospace brake interfaces, while preventing premature failure. Wear track morphologies were examined by FESEM to elucidate mechanisms.

Flexural strength was evaluated by a three-point bending method adapted for disc specimens^24^. Disc-shaped SPS samples (D = 20 mm, thickness = 2 mm) were used without additional machining to avoid altering the sintered microstructure. Support span was set at 16 mm, and fracture strength calculated as:

2 $$\sigma {\mathrm{f}} = {\mathrm{FL}}/\left( {\pi {\mathrm{R}}^{{3}} } \right)$$

where F is fracture load, L is support span, and R is disc radius. Testing was conducted at 0.5 mm/min crosshead speed, with at least five specimens per composition. Thermal conductivity (K) was determined per ASTM E1461-13^25,26^ using:

3 $${\mathrm{K}}\left( {{\mathrm{W}}/{\mathrm{m}} \cdot {\mathrm{k}}} \right) = \alpha \rho C_{p}$$

where α is thermal diffusivity (Xenon flash analysis, Leo model, Germany), ρ is density (kg/m3), and Cp​ is specific heat capacity measured via differential scanning calorimetry (DSC). Tests were performed on 10 × 10 mm2 specimens with 1.4 mm thickness.

## Results and discussions

### XRD patterns of composite before and after sintering

**Fig. 2:**
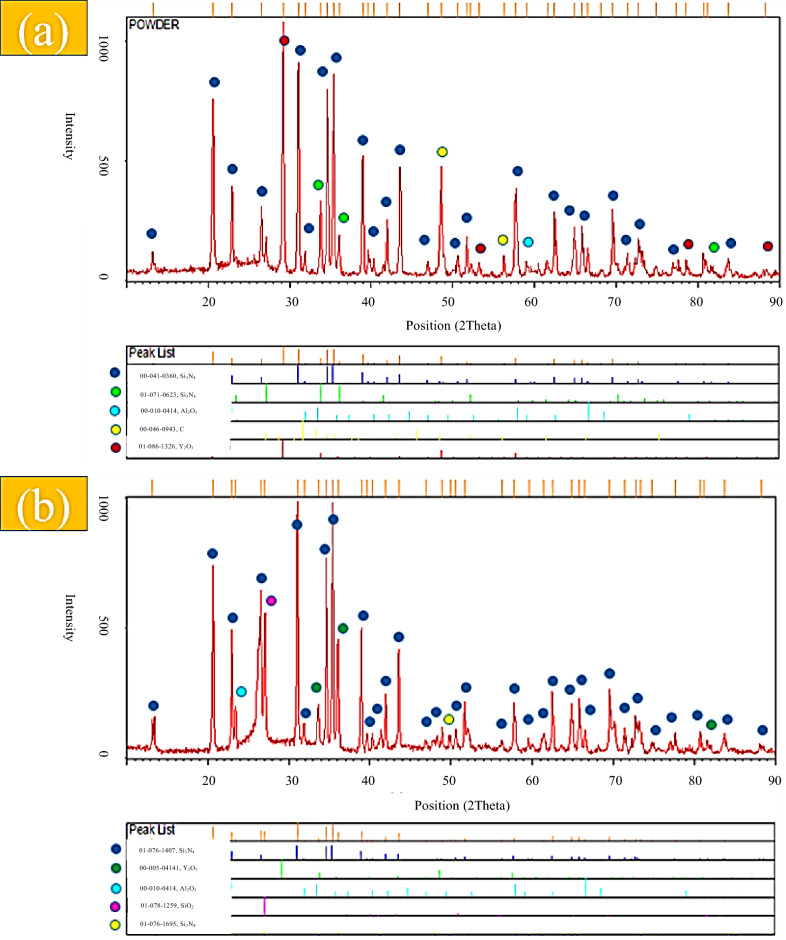
XRD results of C_f_/α-Si_3_N_4_/Al_2_O_3_/Y_2_O_3_ powder, (**a**) pre-sintering (Powder), (**b**) post-sintering.

**Fig. 3:**
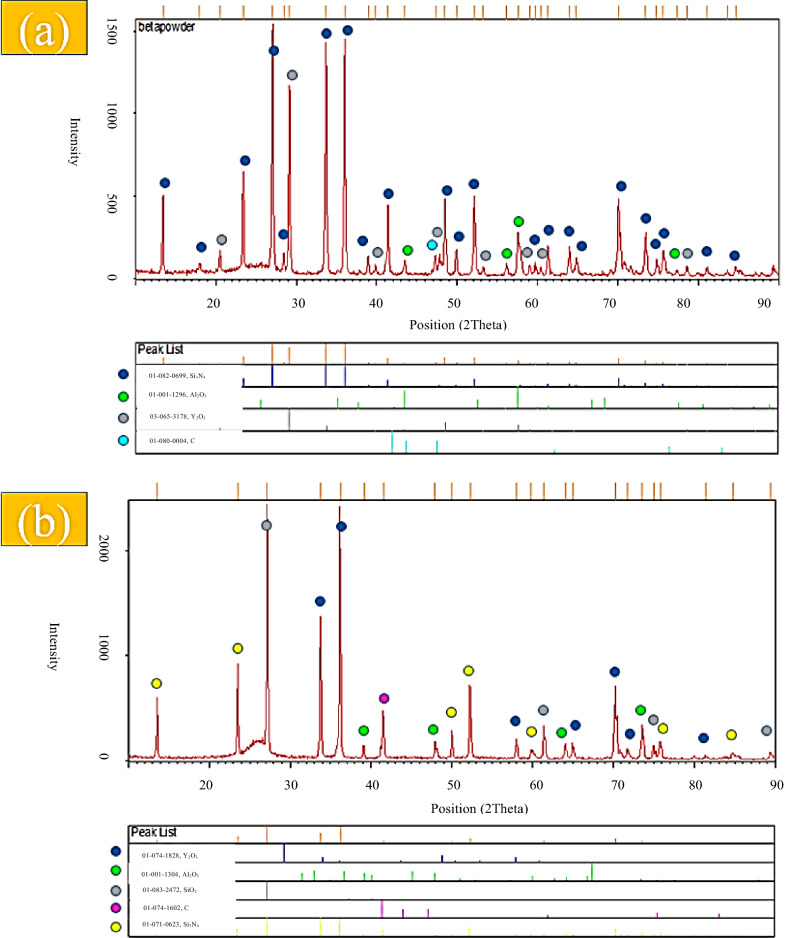
XRD results of C_f_/β-Si_3_N_4_/Al_2_O_3_/Y_2_O_3_ powder, (**a**) pre-sintering (Powder), (**b**) post-sintering.

**Fig. 4:**
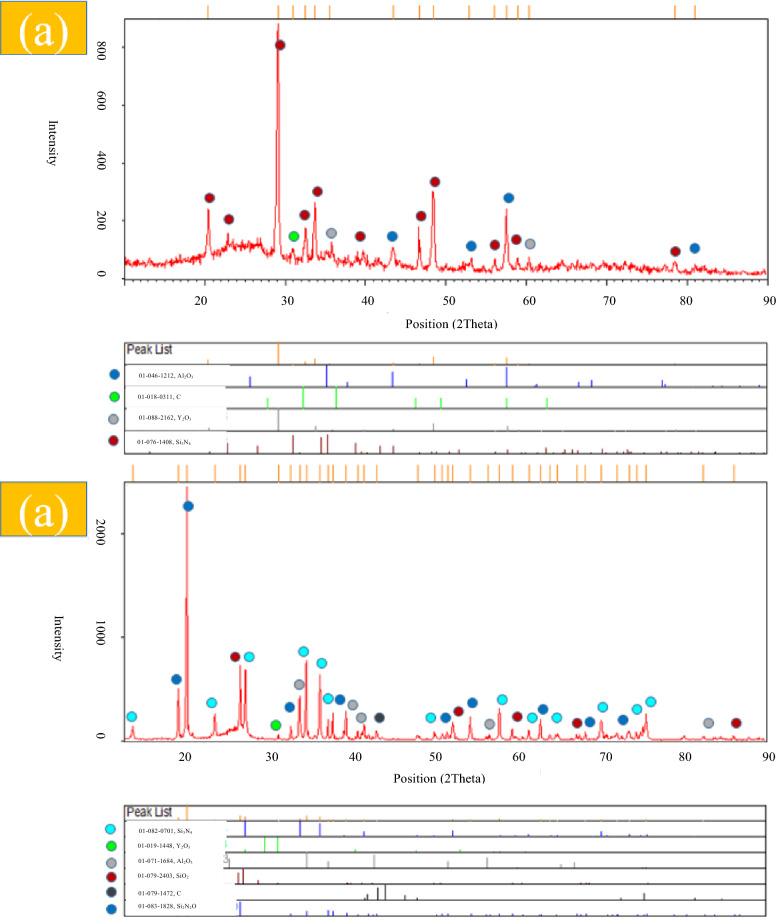
XRD results of C_f_/γ-Si_3_N_4_/Al_2_O_3_/Y_2_O_3_ powder, (**a**) pre-sintering (Powder), (**b**) post-sintering.

XRD patterns of the starting powders (Figs. S1 and S2) confirm the crystalline nature of α- and β-Si_3_N_4_, whereas the commercially labeled γ-Si_3_N_4_ powder exhibits an amorphous structure. Carbon fibers also show no crystalline diffraction peaks, consistent with their turbostratic carbon structure. After spark plasma sintering at 1850 °C (Figs. 2, 3 and 4), all composite samples exhibit significant phase evolution driven by reactions between Si_3_N_4_, surface silica, and the Al_2_O_3_–Y_2_O_3_ sintering additives. In Sample 1 (Cf/α-Si_3_N_4_), the dominant phases after sintering are α- and β-Si_3_N_4_, together with Al_2_O_3_, Y_2_O_3_, and carbon. The appearance of SiO_2_ peaks after sintering (Fig. 2b), absent in the green compact (Fig. 2a), indicates the formation of a silica-rich liquid phase. This liquid originates from the reaction of native SiO_2_ layers on Si_3_N_4_ particles with the oxide additives, facilitating mass transport and densification.

A similar phase assemblage is observed for Sample 2 (C_f_/β-Si_3_N_4_)(Fig. 3a and b) ,where, β-Si_3_N_4_ remains the primary crystalline phase after sintering (Fig. 3b). However, the relative intensity of SiO_2_-related features is higher than in Sample 1, suggesting a larger liquid phase fraction, which is consistent with the lower final density measured for this sample.

In contrast, Sample 3 (C_f_/γ-Si_3_N_4_) exhibits markedly different behavior. Prior to sintering (Fig. 4a), the diffraction pattern confirms the amorphous nature of the γ-Si_3_N_4_ powder. After sintering (Fig. 4b), this amorphous phase fully transforms into crystalline β-Si_3_N_4_ (JCPDS No. 82–0701, hexagonal structure), accompanied by the formation of SiO_2_ and Si₂N₂O secondary phases. The emergence of Si₂N₂O indicates a more extensive reaction between Si_3_N_4_, surface silica, and oxide additives, promoted by the higher chemical reactivity of the amorphous precursor.

Sintering Si_3_N_4_ at 1850 °C under vacuum typically raises concerns regarding thermal decomposition. However, in the present system, the presence of Y_2_O_3_ and Al_2_O_3_ promotes the formation of a transient liquid phase at temperatures as low as ~ 1700 °C, enhancing densification while suppressing decomposition by locally reducing the effective nitrogen partial pressure at particle boundaries. The absence of elemental Si and the lack of excessive SiC formation in the XRD patterns of all sintered samples confirm that Si_3_N_4_ decomposition was effectively mitigated under the applied SPS conditions. Any limited interfacial reaction between carbon fibers and Si_3_N_4_ is expected to result in a thin SiC interlayer, which may be beneficial for interfacial bonding and load transfer.

## Microstructure of sintered bodies

**Fig. 5:**
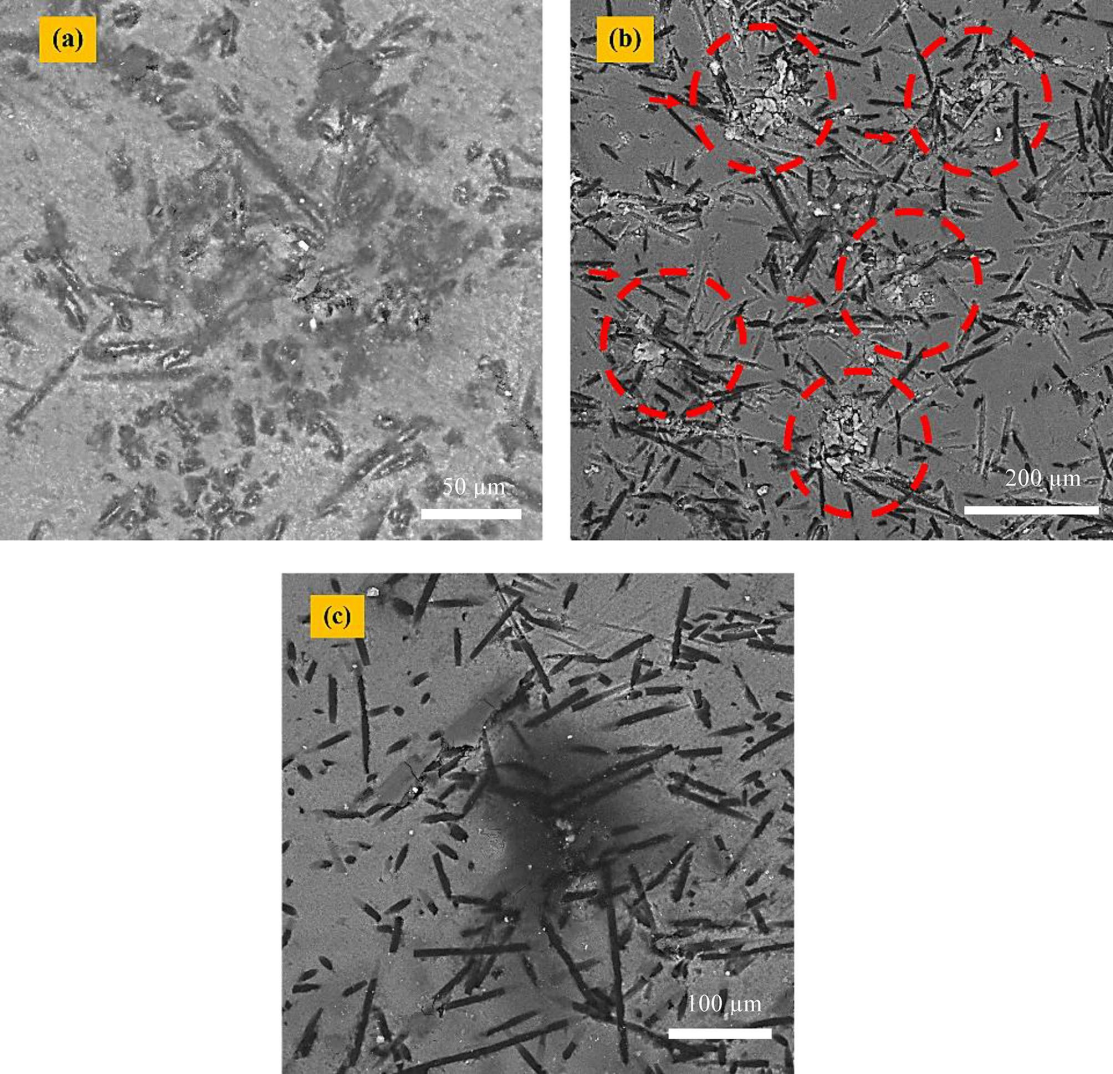
FESEM images related to the sintered samples: (**a**) sample 1, (**b**) sample 2, and (**c**) sample 3.

FESEM micrographs of the sintered composites (Fig. 5) reveal a generally uniform dispersion of carbon fibers within the Si_3_N_4_ matrix for all samples. The originally continuous fibers (5 mm) were shortened during powder processing and consolidation to lengths ranging from approximately 24 to 144 µm, primarily due to high-shear mixing and ultrasonic dispersion.

Among the three compositions, Sample 2 (C_f_/β-Si_3_N_4_) shows localized agglomeration of nanoparticles and carbon fibers, which can negatively affect electrical and thermal conductivity during SPS and hinder uniform densification. In contrast, Samples 1 and 3 exhibit a more homogeneous fiber distribution, which promotes efficient Joule heating and mass transport during sintering, consistent with improved consolidation behavior^27^.

**Fig. 6:**
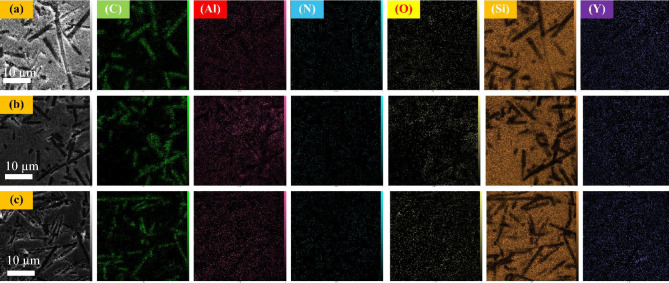
SEM images related to the sintered samples: (**a**) sample 1, (**b**) sample 2, and (**c**) sample 3.

Elemental mapping results (Fig. 6) further confirm the relatively uniform spatial distribution of Si, Al, Y, O, and C within the composite matrices, indicating effective dispersion of sintering additives and carbon fibers across all samples, despite differences in local agglomeration and phase evolution.

## Investigate of density and hardness

The relative densities of the SPS-consolidated composites prepared from α-, β-, and γ-Si_3_N_4_ powders were measured as 96.53%, 90.9%, and 87.47% of the theoretical density, respectively. Accordingly, the residual porosity values were calculated to be 3.47%, 9.1%, and 12.53% for the α-, β-, and γ-based composites.

The observed differences in densification behavior are closely related to the amount and nature of the secondary phases formed during sintering, particularly SiO_2_-based liquid phases. The formation of SiO_2_ during SPS plays a dual role: a limited amount is beneficial by generating a transient liquid phase that enhances mass transport and pore elimination, whereas excessive liquid content can hinder densification and deteriorate mechanical properties. For advanced structural ceramics, the optimal liquid phase content is typically maintained below ~ 5 vol.% to avoid property degradation.

The highest density achieved in the C_f_/α-Si_3_N_4_ composite is attributed to the formation of an optimal liquid phase fraction, which promotes effective particle rearrangement and densification. In contrast, XRD results indicate a higher intensity of SiO_2_-related phases in the C_f_/β-Si_3_N_4_ composite, suggesting an increased liquid volume fraction that likely exceeded the optimal range, leading to reduced final density. For the C_f_/γ-Si_3_N_4_ composite, despite the presence of a well-developed SiO_2_ phase, densification was further impeded by the formation of Si₂N₂O and by the additional energy consumption associated with the crystallization of the initially amorphous γ-Si_3_N_4_ powder during sintering.

Notably, the relative density of the C_f_/α-Si_3_N_4_ composite surpasses that reported for C_f_/SiC brake discs fabricated by the liquid silicon infiltration (LSI) method, which typically achieve only 80–85% of the theoretical density^6,7,27^, highlighting the effectiveness of the SPS route for C_f_/Si_3_N_4_ systems.

Vickers hardness measurements revealed values of 14.1 GPa (VHN = 1433), 13.4 GPa (VHN = 1361), and 20.4 GPa (VHN = 2079) for Samples 1 (α), 2 (β), and 3 (γ), respectively. Despite its lower relative density, the C_f_/γ-Si_3_N_4_ composite exhibited the highest hardness. This behavior is attributed to the finer initial particle size of the γ-Si_3_N_4_ nanopowders and the formation of hard Si₂N₂O secondary phases, which are characterized by strong covalent bonding and inherently high hardness. These results indicate that hardness in the present composites is governed not only by densification level but also by phase constitution and intrinsic bonding characteristics.

## Investigate of coefficient of friction (COF)

Since surface roughness strongly influences tribological behavior, the roughness of all samples was carefully controlled prior to wear testing. The average surface roughness (R_a_) values were measured as 0.36, 0.39, and 0.34 µm for the C_f_/α-Si_3_N_4_, C_f_/β-Si_3_N_4_, and C_f_/γ-Si_3_N_4_ composites, respectively, confirming that all samples possessed comparable surface conditions and that differences in friction and wear behavior primarily originated from intrinsic material properties rather than surface topography.

Wear tests were conducted under a normal load of 5 N, selected based on Hertzian contact stress calculations using Hertzwin software. By considering the elastic moduli of the alumina ball (340 GPa) and the composite disc (110 GPa)^28–31^, along with their respective Poisson’s ratios (0.22 for the ball and 0.085 for the disc)^32–36^, the applied load was determined to generate a contact stress of approximately 1.2 GPa, representative of severe service conditions relevant to aerospace braking systems.

**Fig. 7:**
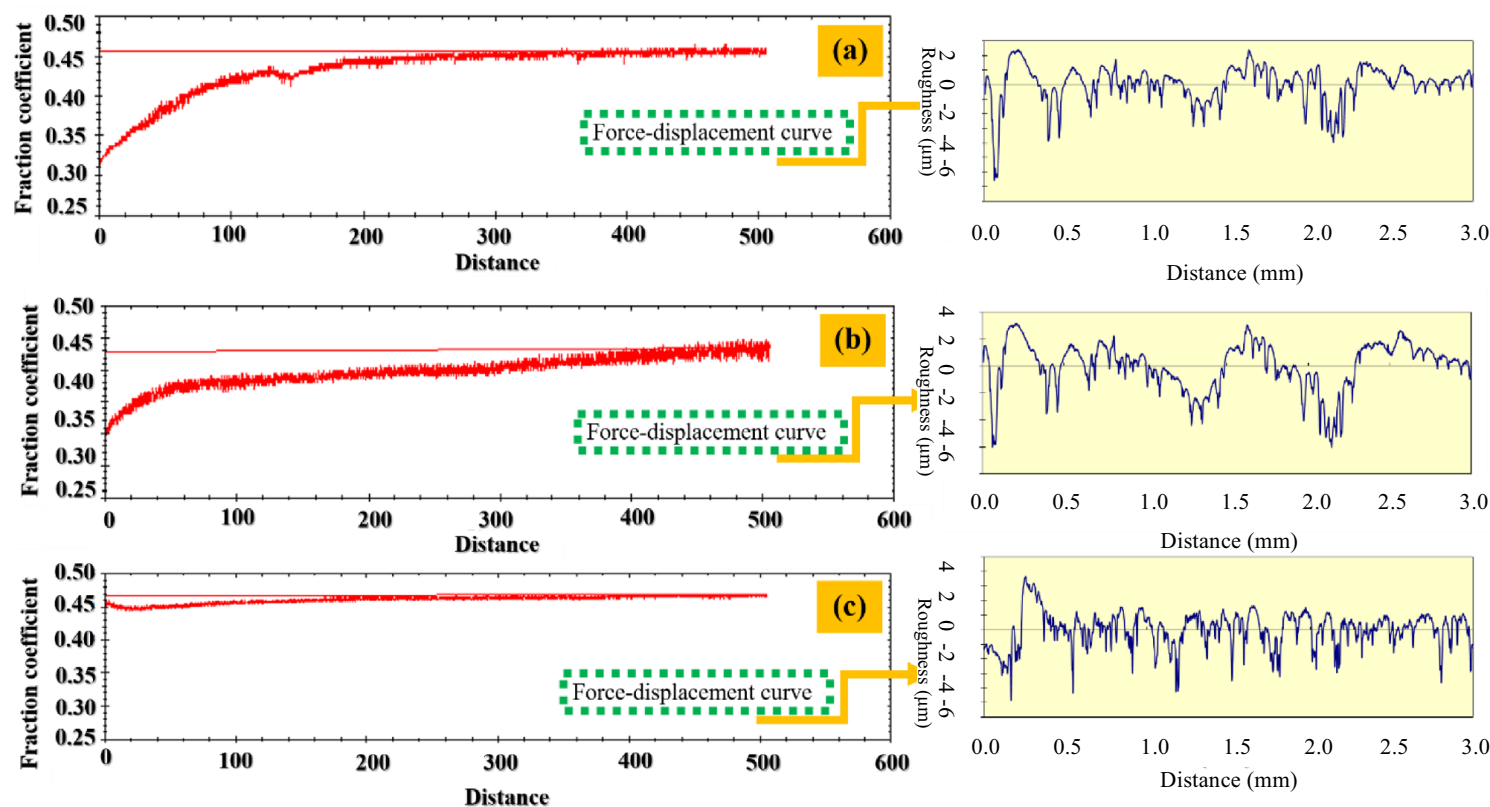
Friction coefficient curves in terms of distance and Ra curves of the sintered samples: (**a**) sample 1, (**b**) sample 2, and (**c**) sample 3.

The measured coefficients of friction (Fig. 7) for the C_f_/α-, C_f_/β-, and C_f_/γ-Si_3_N_4_ composites were approximately 0.46, 0.48, and 0.44, respectively. Notably, the C_f_/γ-Si_3_N_4_ composite exhibited a COF closest to that reported for aerial brake discs (~ 0.4), whereas the C_f_/α-Si_3_N_4_ sample demonstrated the most stable friction response, with minimal fluctuations throughout the test. This stability indicates the formation of a robust and continuous tribofilm at the sliding interface.

Wear rate measurements further differentiate the tribological performance of the composites. The wear rates of Samples 1 (α), 2 (β), and 3 (γ) were determined to be on the order of 10⁻⁷ g/N·m, with the C_f_/α-Si_3_N_4_ composite consistently exhibiting the lowest wear rate and the most stable friction behavior. This superior performance is attributed to its optimized microstructure and the effective self-lubricating action provided by uniformly distributed carbon fibers.

**Fig. 8:**
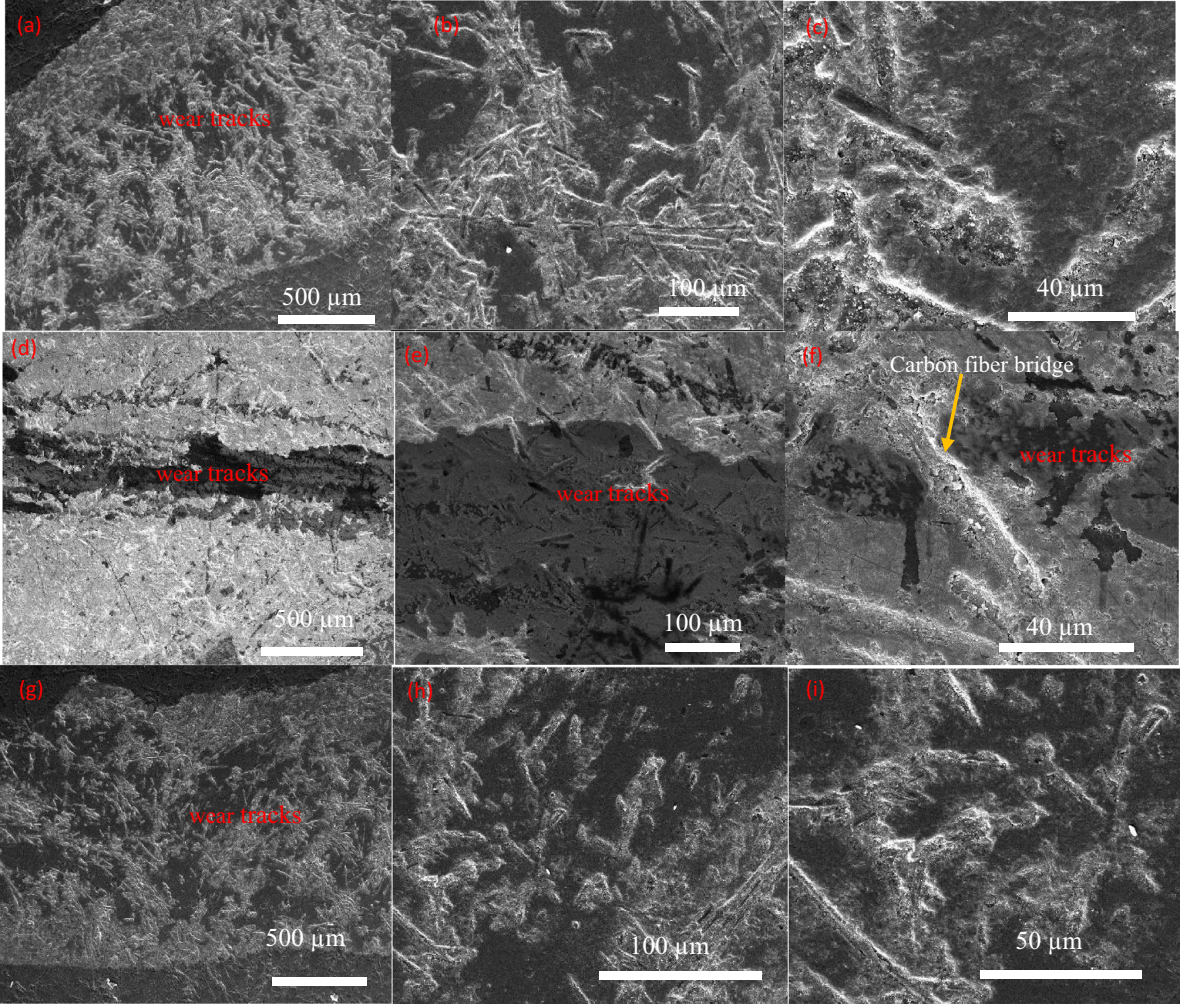
FESEM images of wear tracks (**a**–**c**) sample 1, (**d**–**f**) sample 2, and (**g**–**i**) sample 3.

SEM observations of the wear tracks (Fig. 8) and corresponding EDS mapping (Fig. S6) reveal that mild abrasive wear is the dominant wear mechanism for all composites, characterized by shallow grooves along the sliding direction. However, the key distinction of the C_f_/α-Si_3_N_4_ composite lies in the formation of a protective tribofilm composed primarily of smeared carbonaceous material and tribo-oxidation products. This tribofilm acts as a lubricating layer, reducing direct asperity contact between the composite and the alumina counterface and thereby stabilizing the COF at approximately 0.46.

In addition to tribofilm formation, carbon fiber bridging was frequently observed within the wear tracks of the C_f_/α-Si_3_N_4_ composite. Individual fibers span microcracks and voids generated during sliding, effectively suppressing crack propagation and mitigating material removal. This mechanism, combined with the in-situ formation of an interlocking, needle-like β-Si_3_N_4_ grain structure, enhances crack deflection and energy dissipation, resulting in improved wear resistance despite partial fiber shortening (24–144 µm) caused by high-shear mixing and SPS consolidation.

By contrast, the C_f_/γ-Si_3_N_4_ composite, although exhibiting higher bulk hardness, shows a more brittle wear response. Its higher residual porosity and the presence of brittle secondary phases promote subsurface cracking and deeper wear grooves, leading to increased material removal. The C_f_/β-Si_3_N_4_ composite displays intermediate tribological performance, lacking both the optimal self-reinforced grain morphology and the uniform fiber distribution required for superior wear resistance.

Overall, the outstanding tribological performance of the C_f_/α-Si_3_N_4_ composite arises from the synergistic interaction of a tough, self-reinforced matrix, the formation of a stable carbon-rich tribofilm, and effective carbon fiber bridging. This multi-mechanism synergy enables stable friction behavior and reduced wear under high contact stress, making the α-Si_3_N_4_-derived composite a strong candidate for demanding aerospace braking applications. The observed tribofilm stability and damage-tolerant wear mechanisms are consistent with previous reports on C_f_/SiC and elongated-grain Si_3_N_4_-based composites^1,5,37–39^.

The processing route, notably the high-shear mixing and high-pressure SPS consolidation, may induce some shortening and damage to the carbon fibers, as suggested by the reduced fiber lengths (24–144 μm) observed post-sintering compared to the initial 5 mm. While such microstructural alterations could potentially diminish the fiber-bridging contribution to fracture toughness, the dominant effect in the α-Si_3_N_4_-derived composite appears to be the positive formation of an interlocking, self-reinforced β-Si_3_N_4_ grain network. This in-situ toughened matrix, coupled with residual fiber pull-out mechanisms evident in fracture surfaces (Fig. 8), effectively counterbalances any negative impact from fiber processing damage, leading to the superior overall mechanical and tribological performance observed.

## Investigation of flexural strength and fracture toughness

**Fig. 9:**
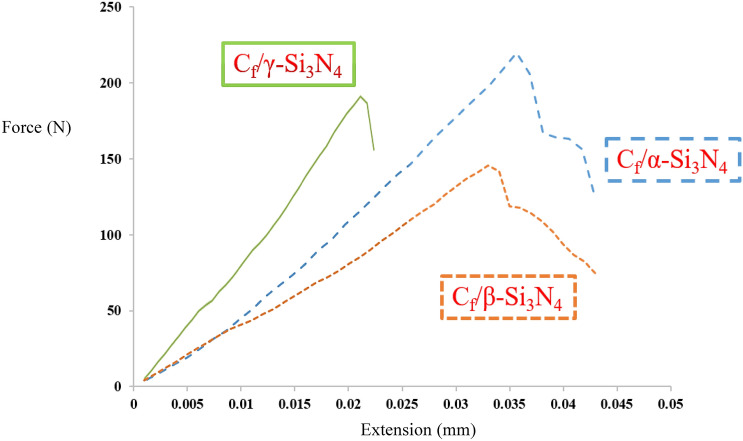
Force–displacement curve of the sintered samples with different Si3N4 phases.

The flexural strength results indicate that all composites exhibited predominantly brittle fracture behavior, as failure occurred within the elastic region of the stress–strain response. The measured flexural strengths of the C_f_/α-Si_3_N_4_, C_f_/β-Si_3_N_4_, and C_f_/γ-Si_3_N_4_ composites were 226 ± 3, 142 ± 4, and 194 ± 4 MPa, respectively (Fig. 9).

**Fig. 10:**
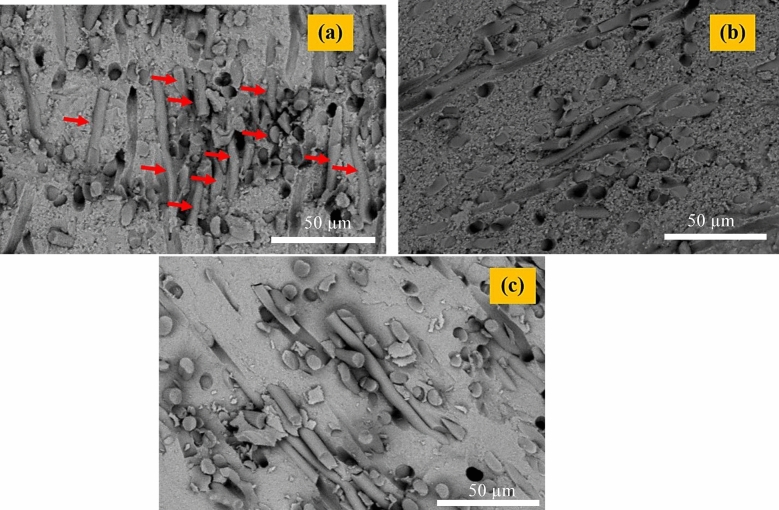
SEM images of the fracture surface of the sintered samples after flexural strength test: (**a**) sample 1, (**b**) sample 2, and (**c**) sample 3.

The superior flexural strength of the C_f_/α-Si_3_N_4_ composite (Sample 1) is primarily attributed to its unique post-sintering microstructure and high relative density. As revealed by FESEM observations (Figs. 8 and 10) and quantitative phase analysis using MAUD software, this composite consists predominantly of elongated, needle-like β-Si_3_N_4_ grains (~ 79%) embedded within a minor fraction of equiaxed α-Si_3_N_4_ grains (~ 20%). These elongated β-Si_3_N_4_ grains act as an in-situ self-reinforcement phase, analogous to whiskers, effectively impeding crack initiation and propagation. Their interlocking morphology increases crack path tortuosity and enhances load transfer across the matrix, thereby improving flexural strength.

In addition to microstructural reinforcement, density plays a critical role in determining flexural strength. Higher density corresponds to lower residual porosity, which reduces stress concentration sites and delays crack initiation. The C_f_/α-Si_3_N_4_ composite exhibited the highest relative density among all samples, consistent with its superior flexural strength. Interestingly, the C_f_/γ-Si_3_N_4_ composite demonstrated a higher flexural strength than the C_f_/β-Si_3_N_4_ composite despite its lower density. This behavior is attributed to the presence of the Si₂N₂O phase in the γ-derived composite, which is characterized by strong covalent bonding and contributes to enhanced local stiffness and strength.

Fracture surface analyses (Fig. 10) further reveal that fiber pull-out is a dominant toughening mechanism in all composites. The presence of pulled-out carbon fibers indicates effective load transfer across the fiber–matrix interface and contributes to energy dissipation during fracture, thereby enhancing both strength and toughness.

The fracture toughness (*K*_IC_​) values of Samples 1, 2, and 3 were measured as 10.87 ± 0.6, 8.36 ± 0.3, and 4.91 ± 0.5 MPa m^0.5^, respectively. The significantly higher fracture toughness of the C_f_/α-Si_3_N_4_ composite is attributed to a combination of its high relative density and its self-reinforced microstructure. In Si_3_N_4_-based ceramics, fracture toughness is strongly governed by microstructural features, and mechanisms such as grain bridging, fiber pull-out, crack deflection, and crack branching play decisive roles. The elongated β-Si_3_N_4_ grains in Sample 1 actively participate in these mechanisms, promoting crack shielding and increasing fracture resistance.

A direct comparison with literature values for ceramic matrix composites must consider the manufacturing route and testing methodology. The indentation fracture resistance of the optimal Cf/α-Si_3_N_4_ composite (~ 10.9 MPa m^0.5^) is competitive with or exceeds that reported for many C_f_/SiC composites fabricated via Chemical Vapor Infiltration (CVI), which typically range from 5 to 7 MPa m^0.5^^7,14,40,41^. It is noted, however, that C_f_/SiC composites produced by Polymer Impregnation and Pyrolysis (PIP)^42,43^ can achieve higher fracture toughness (15–18 MPa m^0.5^). The significance of the present work lies not in surpassing all existing systems, but in demonstrating that a carefully selected Si_3_N_4_ polymorph (α-phase) processed via a rapid SPS route can yield a composite with a well-balanced property set—combining respectable fracture resistance, good thermal conductivity, and stable tribological behavior—thereby offering a promising alternative fabrication strategy for tailored thermostructural components.

The superior flexural strength and fracture toughness observed in the C_f_/α-Si_3_N_4_ composite (Sample 1) can be attributed to a synergistic effect of high relative density and a unique self-reinforced microstructure. The in-situ formation of interlocking, needle-like β-Si_3_N_4_ grains within the α-Si_3_N_4_ matrix, as confirmed by XRD and FESEM, effectively promotes toughening mechanisms such as crack deflection and fiber pull-out. This microstructure acts similarly to whisker-reinforced composites, where the elongated grains bridge cracks and increase the fracture path complexity. Consequently, the achieved *K*_IC_ ​ value of 10.87 MPa m^0.5^ not only surpasses that of our other samples but also exceeds the performance of many C_f_/SiC composites manufactured by conventional methods like CVI, as referenced in^41,42^. This demonstrates the potential of SPS with an α-Si_3_N_4_ starting powder to create intrinsically toughened composites without the need for external whiskers^44–48^.

## Investigate of thermal conductivity

The thermal conductivity coefficients of the C_f_/Si_3_N_4_ composites, summarized in Table 1, were measured as 0.0156, 0.0131, and 0.0144 cm^2^ s^−1^ for Sample 1 (C_f_/α-Si_3_N_4_), Sample 2 (C_f_/β-Si_3_N_4_), and Sample 3 (C_f_/γ-Si_3_N_4_), respectively. These values correspond to thermal conductivities of 66, 58, and 53 W m^−1^ K^−1^.

**Table 1:** Thermal diffusivity (α) parameter measured by XFA method for different samples measeared according to ASTM E1461.

No. (sample 1)	α (cm^2^/s)	No. (sample 2)	α (cm^2^/s)	No. (sample 3)	α (cm^2^/s)
1	0.0150	1	0.0145	1	0.0144
2	0.0143	2	0.0107	2	0.0145
3	0.0147	3	0.0107	3	0.0133
4	0.0176	4	0.0188	4	0.0150
5	0.0168	5	0.0108	5	0.0149
Average	0.0156	Average	0.0131	Average	0.0144

A clear correlation between thermal conductivity, relative density, and microstructural integrity is observed. The highest thermal conductivity is achieved in the C_f_/α-Si_3_N_4_ composite, which also exhibits the highest relative density and lowest porosity. Reduced porosity minimizes phonon scattering at pore surfaces and grain boundaries, thereby facilitating more efficient heat transport through the ceramic matrix. In contrast, the lower thermal conductivity of the C_f_/γ-Si_3_N_4_ composite is primarily attributed to its higher residual porosity and reduced densification, which introduce additional phonon scattering centers.

The absolute thermal conductivity values of the C_f_/Si_3_N_4_ composites are lower than those of metallic brake discs; however, they fall within the optimal range for ceramic brake materials. Excessively high thermal conductivity can result in rapid heat dissipation away from the friction interface, potentially reducing braking efficiency, whereas overly low conductivity can cause localized heat accumulation, thermal gradients, and an increased risk of thermal cracking. From this perspective, the moderate thermal conductivity of the C_f_/α-Si_3_N_4_ composite represents a balanced thermal management capability.

The reduced thermal conductivity of the composites relative to monolithic Si_3_N_4_ is also influenced by the incorporation of carbon fibers, which possess a comparatively low thermal conductivity (~ 3 W m^−1^ K^−1^)^26,49,50^. Furthermore, the C_f_/Si_3_N_4_ interfaces act as effective phonon barriers due to acoustic mismatch, further limiting heat transport across the composite. Notably, the thermal conductivity of the C_f_/γ-Si_3_N_4_ composite is comparable to that reported for Cf/SiC brake discs fabricated via the CVI process^46,47^, indicating that C_f_/Si_3_N_4_ composites processed by SPS can meet the thermal performance requirements of aerospace braking systems.

Overall, the combination of moderate thermal conductivity and high specific heat capacity in the C_f_/α-Si_3_N_4_ composite suggests an enhanced ability to absorb, distribute, and manage frictional heat during braking. This thermal behavior is particularly advantageous for aerospace applications, where resistance to thermal fade and stable performance under severe thermal loading are critical safety requirements.

## Quantitative microstructural analysis and toughening mechanisms

While qualitative FESEM observations revealed the presence of elongated, needle‑like β‑Si_3_N_4_ grains in the α‑phase‑derived composite (Sample 1), a quantitative microstructural analysis was conducted to substantiate their role in enhancing fracture toughness. Image analysis of FESEM micrographs was performed using ImageJ software to determine the aspect ratio and volume fraction of the elongated β‑Si_3_N_4_ grains formed in situ during SPS.

The analysis revealed an average grain aspect ratio exceeding 5 and a volume fraction of approximately 30–40% for the elongated β‑Si_3_N_4_ grains in Sample 1. This high fraction of high‑aspect‑ratio grains provides a direct, quantitative explanation for the superior fracture toughness (10.87 MPa·m⁰·^5^) observed in this composite. Unlike Samples 2 and 3, which lack a comparable self‑reinforced microstructure, Sample 1 benefits from multiple, simultaneously active toughening mechanisms that operate across different length scales.

The enhanced fracture resistance can be attributed to the following quantitatively supported mechanisms:

I. Crack Deflection

The randomly oriented, high‑aspect‑ratio β‑Si_3_N_4_ grains force propagating cracks to repeatedly change direction, significantly increasing crack path tortuosity and the energy required for crack propagation.

II. Crack Bridging

Elongated β‑Si_3_N_4_ grains bridge the crack wake and apply closing stresses that shield the crack tip from the full applied load, thereby delaying catastrophic failure.

III. Synergy with Fiber Pull‑out

The self‑reinforced ceramic matrix operates synergistically with the carbon fiber reinforcement. While the fibers provide macroscopic energy dissipation through pull‑out and debonding, the elongated β‑Si_3_N_4_ grains contribute microscale crack shielding, resulting in a highly effective multi‑scale toughening system.

This quantitative microstructural evidence transforms the interpretation of the mechanical results from a simple correlation to a clear causative relationship. It firmly establishes that the α → β phase transformation during SPS is not merely a crystallographic change, but a powerful microstructural engineering strategy for designing intrinsically toughened C_f_/Si_3_N_4_ composites without the need for external whisker reinforcements.

## Conclusion

This study demonstrates that the initial phase of Si_3_N_4_ powder plays a decisive role in controlling the microstructural evolution and resulting properties of C_f_/Si_3_N_4_ composites fabricated by spark plasma sintering. Among the investigated precursors, α-Si_3_N_4_ enabled the formation of an in-situ self-reinforced β-Si_3_N_4_ grain network, leading to an optimal balance of fracture toughness (10.87 MPa m^0.5^), thermal conductivity (66 W/m K), and stable tribological behavior (COF ≈ 0.46).

The superior performance of the α-derived composite is attributed to the synergistic interaction between the elongated β-Si_3_N_4_ grains and carbon fiber reinforcement, which promotes crack deflection, crack bridging, and the formation of a stable tribofilm during sliding. In contrast, composites derived from β- and γ-Si_3_N_4_ powders exhibited inferior densification or excessive hardness without a corresponding improvement in overall performance.

These findings establish a phase-informed microstructural design strategy for C_f_/Si_3_N_4_ composites, demonstrating that controlled α → β transformation during SPS can be effectively exploited as a microstructural engineering tool. The insights gained in this study provide a foundation for developing high-performance non-oxide ceramic composites for demanding thermostructural applications such as aerospace brake.

## Supplementary Information

Supplementary Information.

## Data Availability

The datasets generated and analyzed during the current study are available from the corresponding author upon reasonable request.
