# Supplementary material for: Concurrent optimization of fracture toughness, thermal conductivity, and tribological behavior in Cf/Si3N4 composites via phase driven selection
Source: Sci Rep. 2026 Mar 28;16:10739. doi: 10.1038/s41598-026-44244-7 (PMC13039794; doi:10.1038/s41598-026-44244-7)
Supplement: Supplementary file 1 — Supplementary Information. [file 41598_2026_44244_MOESM1_ESM.pdf]

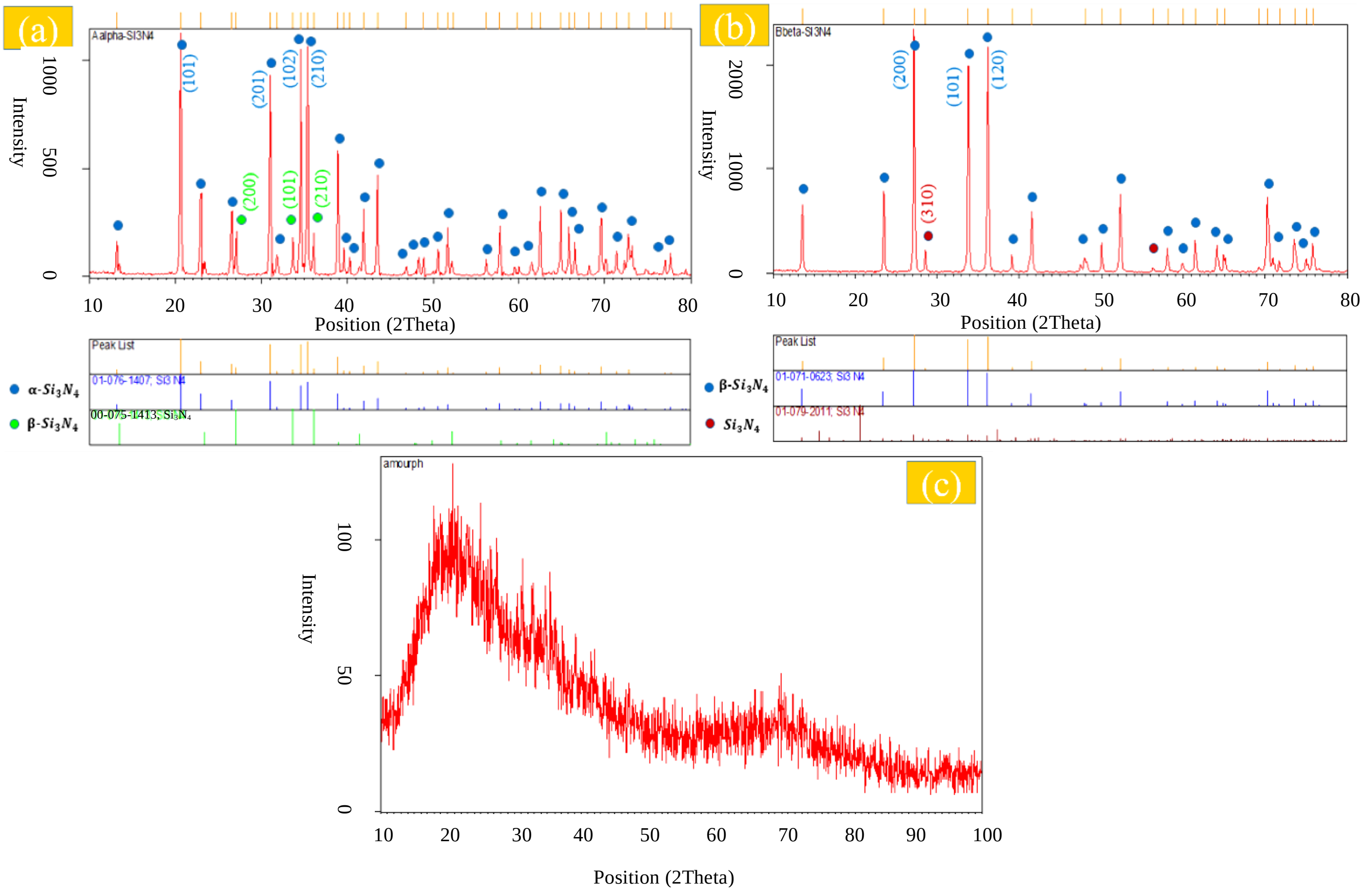

Fig. S1. XRD results of (a) α-Si_3N_4 powder (b) β-Si_3N_4 powder (c) γ-Si_3N_4 powder.

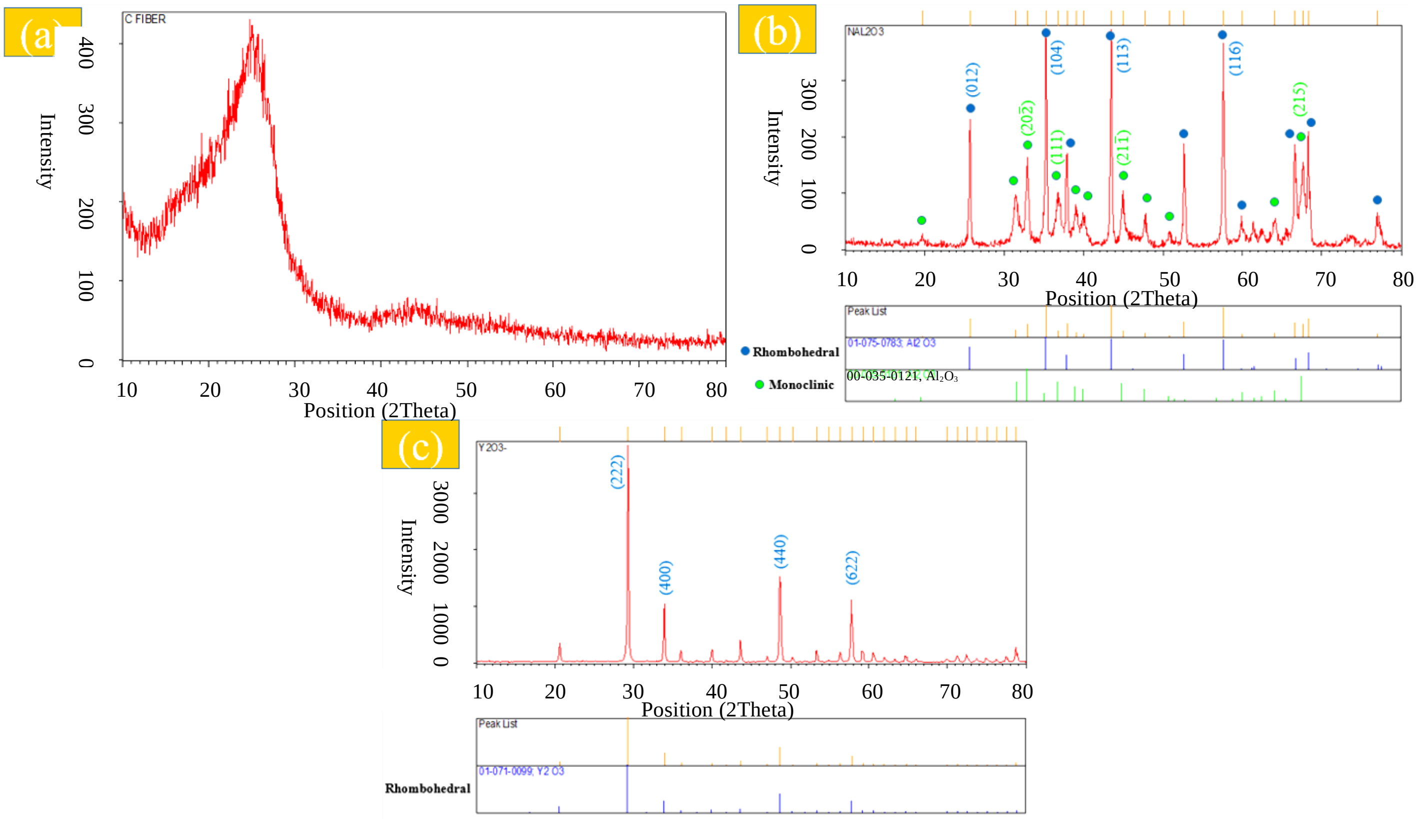

Fig. S2. XRD results of (a) carbon fiber (b) Al_2O_3 powder (c) Y_2O_3 powder.

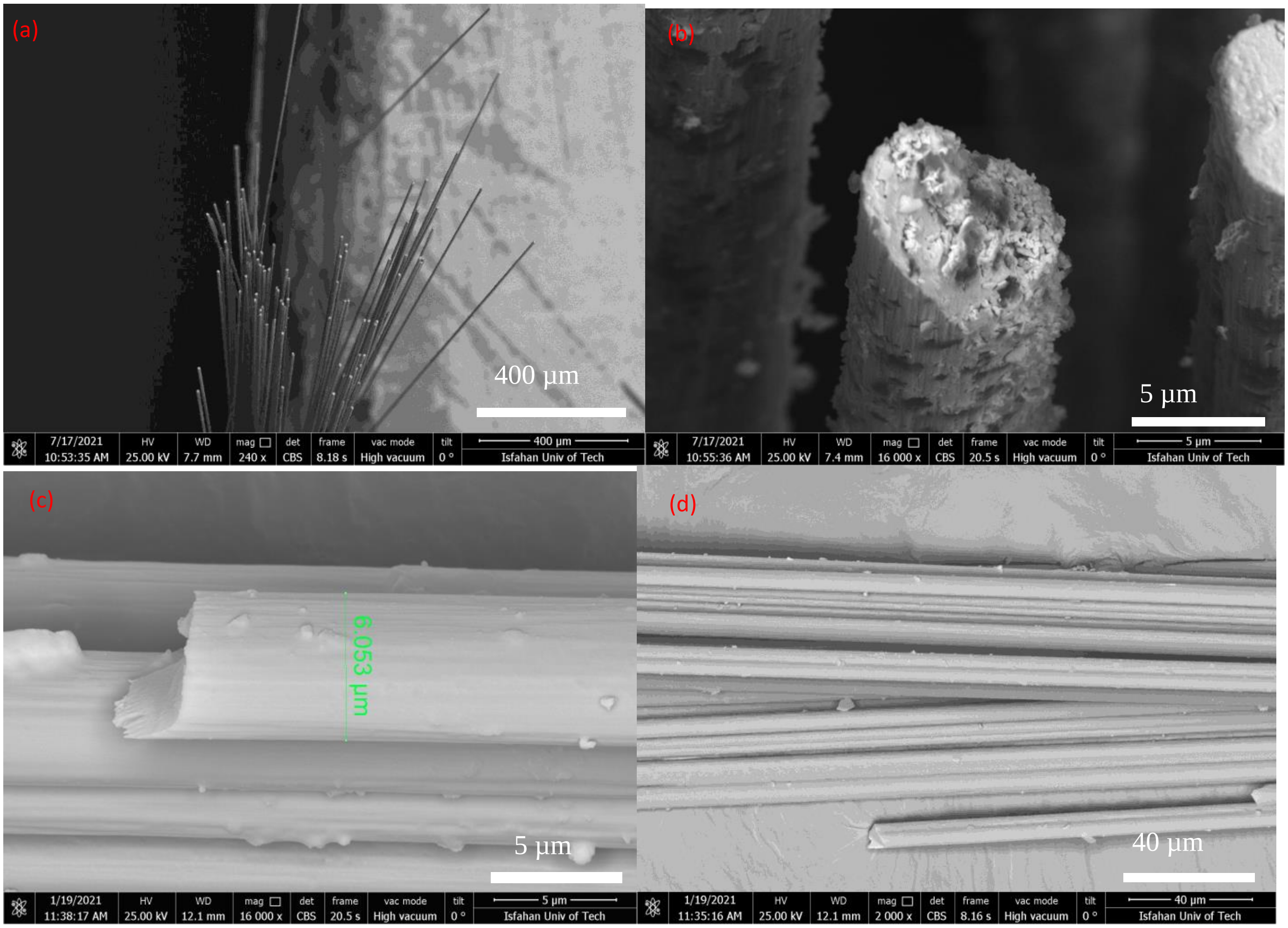

Fig S3: FE-SEM images of carbon fiber secondary mode (a) back-scattered mode (b) with different magnifications

(a)

Counts

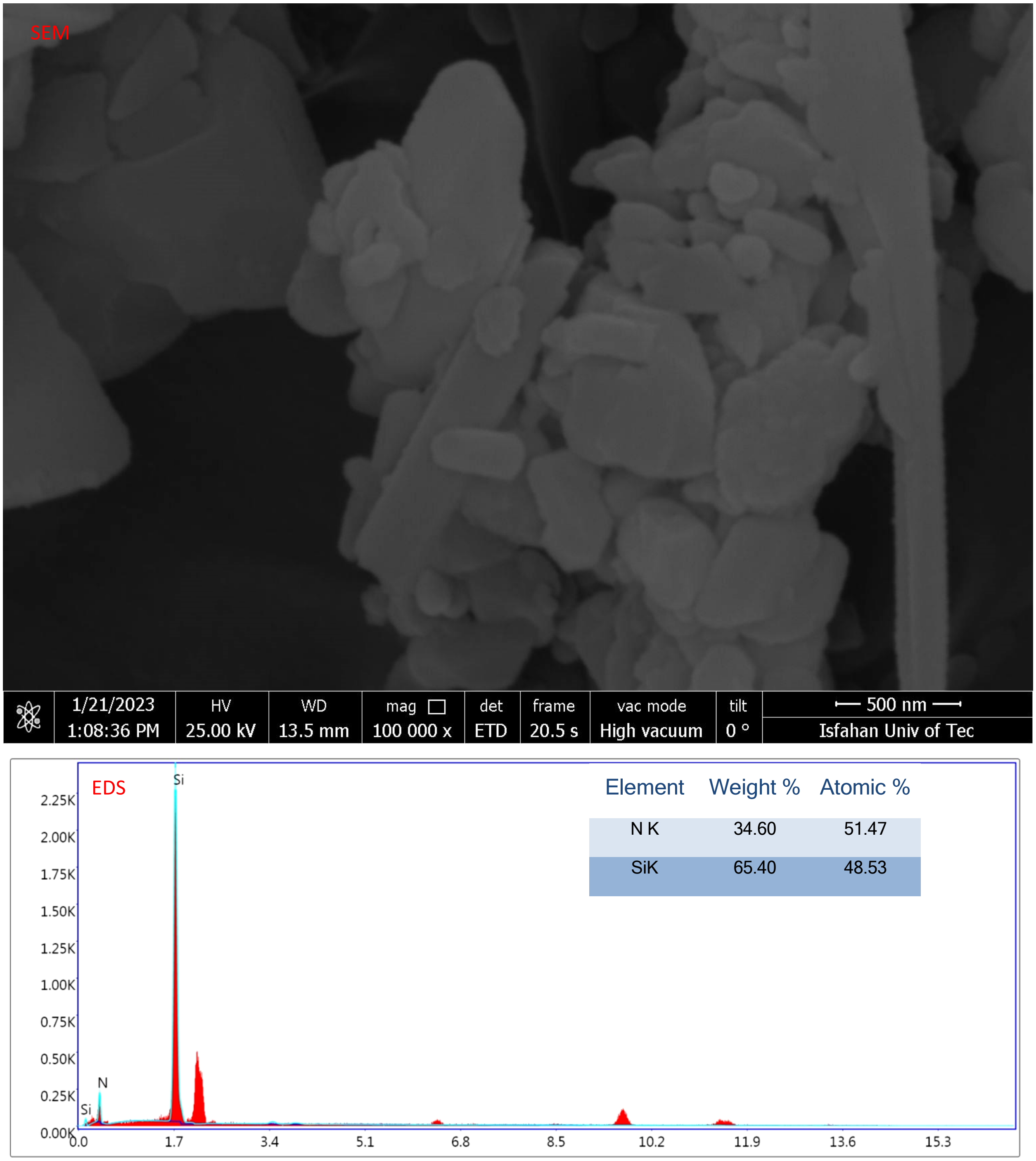

Energy (KeV)

Fig S4: SEM and EDS analysis of Si_3N_4 powder with different phases (a)α- Si_3N_4

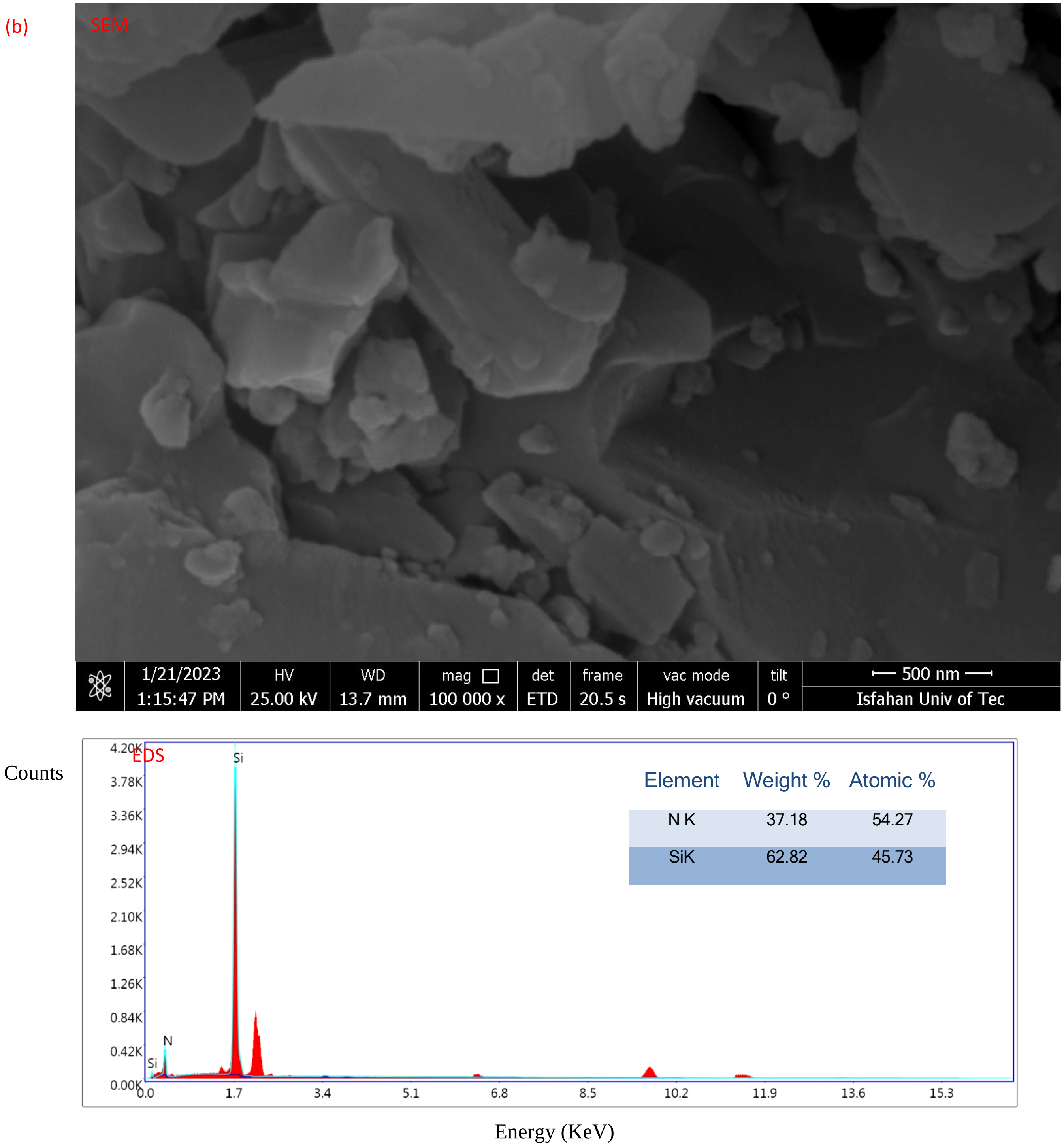

Fig S4: SEM and EDS analysis of Si_3N_4 powder with different phases (b)β- Si_3N_4

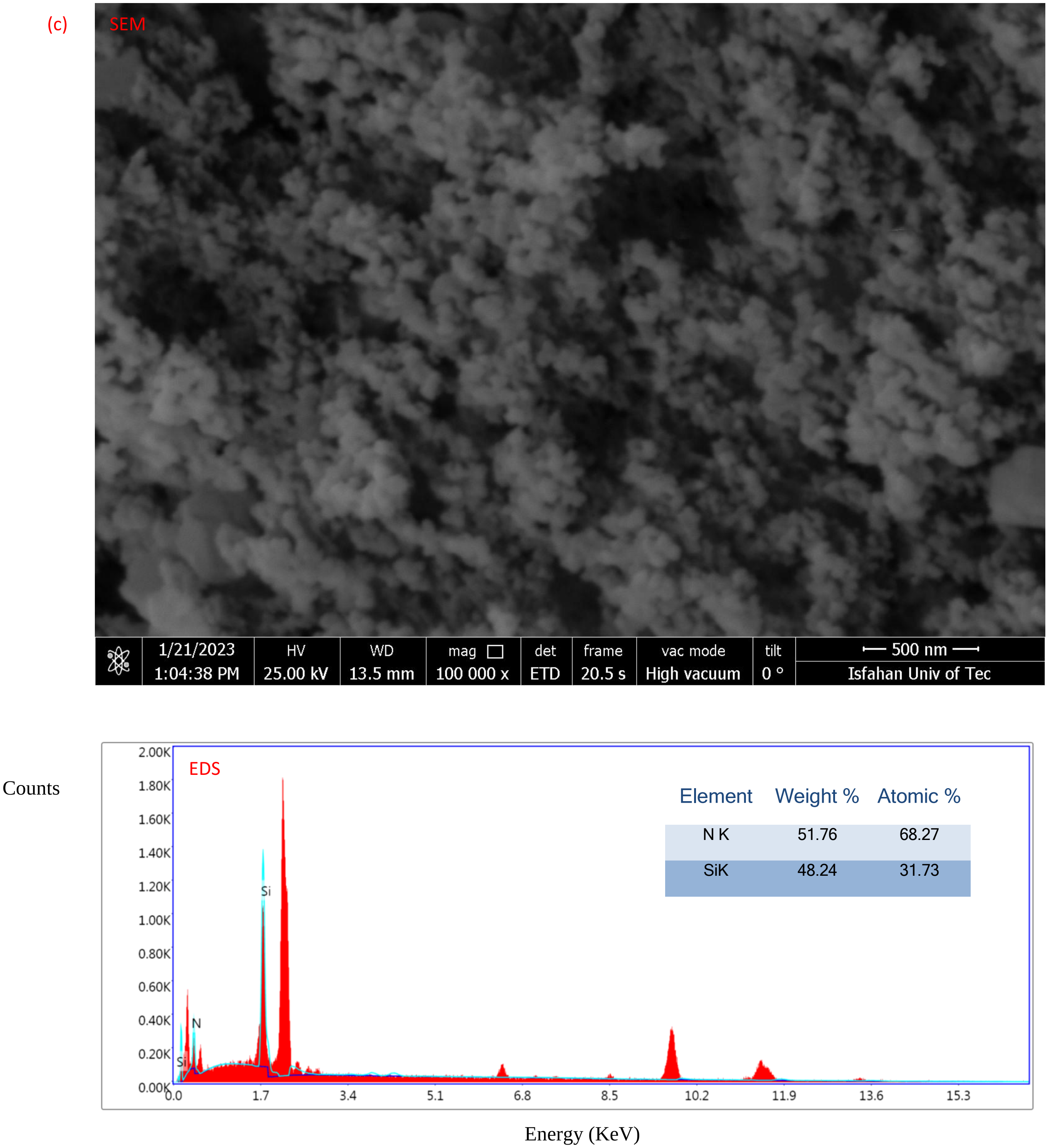

Fig S4: SEM and EDS analysis of Si_3N_4 powder with different phases (c) γ- Si_3N_4

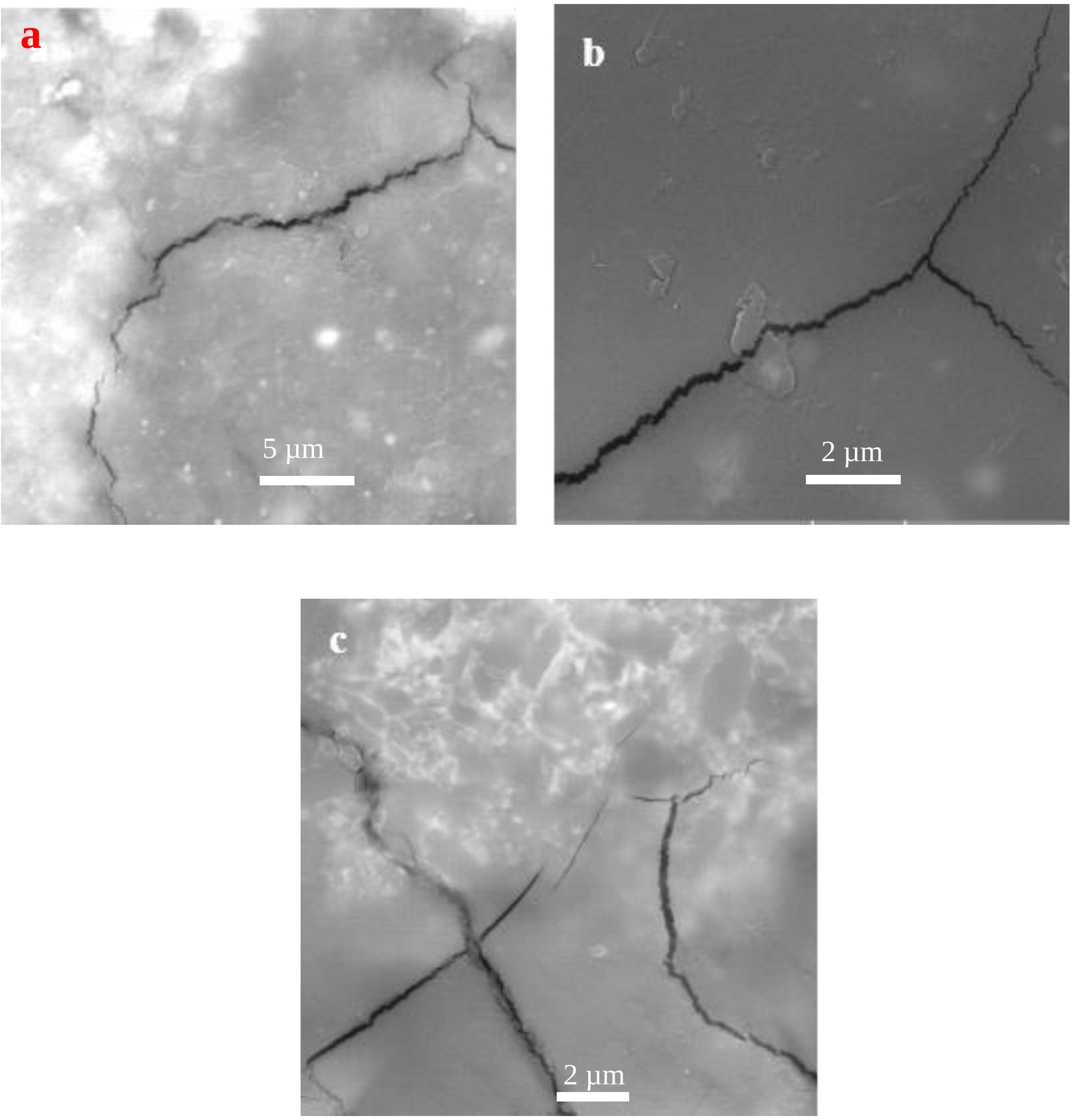

Fig. S5. SEM images of the crack created by the Vickers indenter on the sintered samples: a) sample 1, b) sample 2, c) sample 3

(a)

(b)

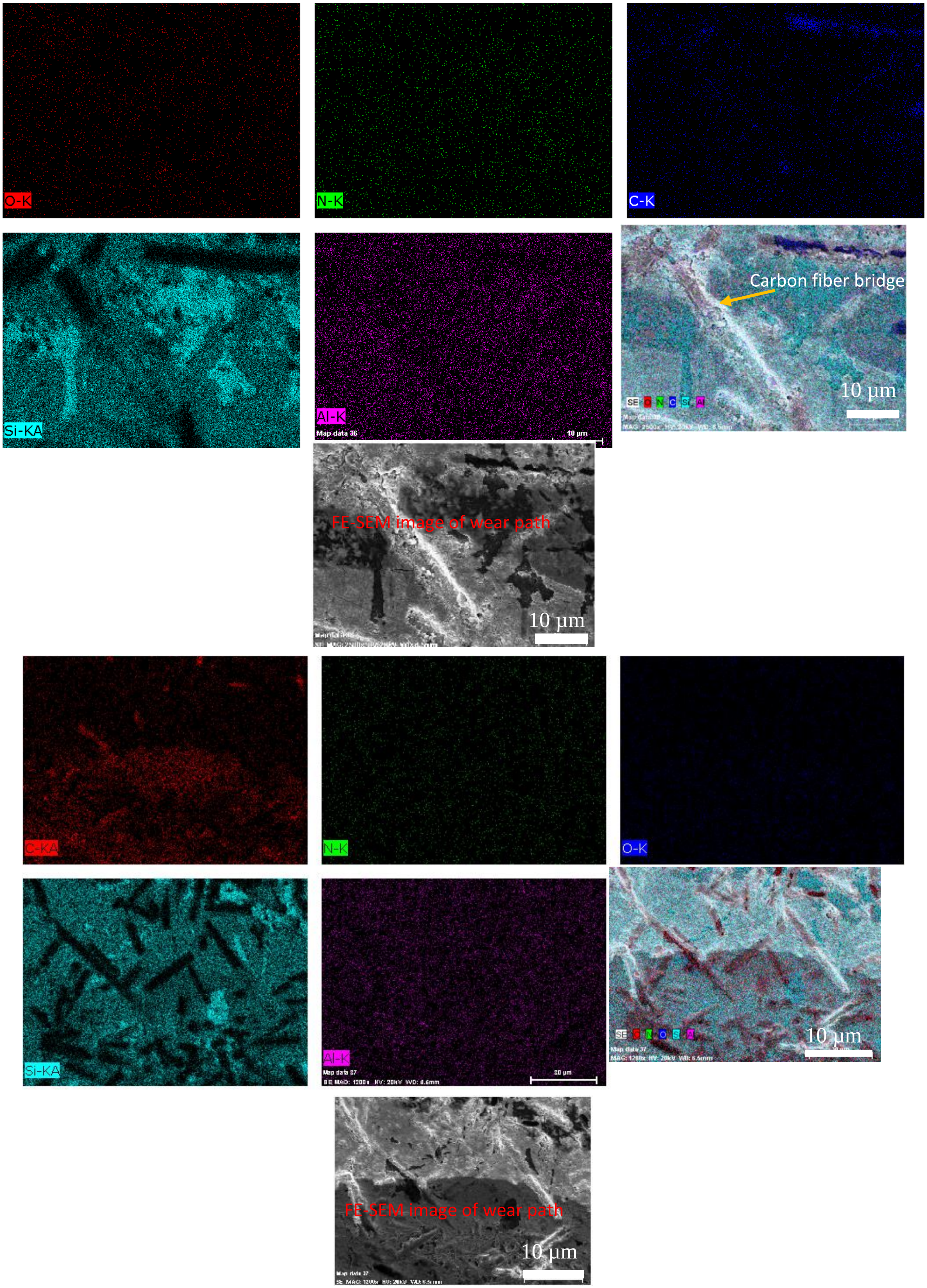
O-K
N-K
C-K
Si-KA
Al-K
Carbon fiber bridge
10 μm
FE-SEM image of wear path
10 μm
C-KA
N-K
O-K
Si-KA
Al-K
10 μm
FE-SEM image of wear path
10 μm

(c)

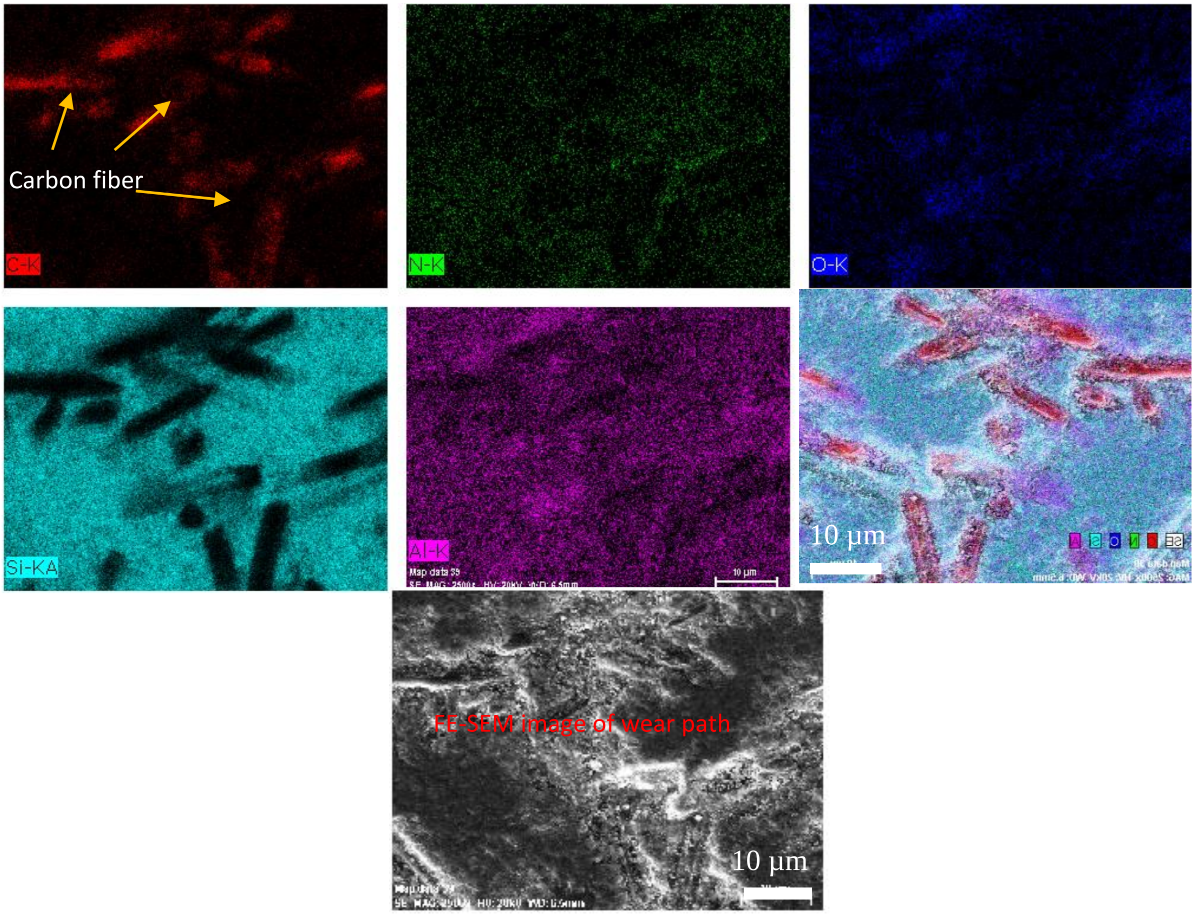

Fig. S6. EDS mapping of wear path a) sample 1, b) sample 2, and c) sample 3.
